# Evaluación del UMELISA CHAGAS^®^ con la incorporación de nuevos péptidos monoméricos y quiméricos representativos de diferentes regiones de *Trypanosoma cruzi*

**DOI:** 10.7705/biomedica.5435

**Published:** 2021-05-31

**Authors:** Idialis Hernández, Milenen Hernández, Jeny González, Ivonne Gómez, Orlando Zulueta, Grisell Ramos, Darien Ortega, Dunia Clara Bequer, Giosvany Ernesto Martínez

**Affiliations:** 1 Aurora Delahanty Centro de Inmunoensayo, La Habana, Cuba Centro de Inmunoensayo La Habana Cuba

**Keywords:** Trypanosoma cruzi, enfermedad de Chagas/diagnóstico, anticuerpos, péptidos, Trypanosoma cruzi, Chagas disease/diagnosis, antibodies, peptides

## Abstract

**Introducción.:**

La mayoría de las personas con enfermedad de Chagas desarrolla anticuerpos específicos contra *Trypanosoma cruzi.* En la infección temprana se producen anticuerpos IgM contra *T. cruzi* que son reemplazados por IgG durante el curso de la enfermedad. Los primeros síntomas de la enfermedad suelen ser muy leves y atípicos, por lo que a menudo no se detecta en la fase aguda.

**Objetivos.:**

Evaluar la sensibilidad y la especificidad clínica y analítica, la precisión y la eficacia del UMELISA CHAGAS^®^ con la incorporación de nuevos péptidos sintéticos en la fase sólida representativos de la proteína SAPA *(Shed Acute Phase Antigen)* y del antígeno TSA *(Trypomastigote Surface Antigen).*

**Materiales y métodos.:**

Se evaluó un panel de desempeño de título mixto anti-I *cruzi* y uno de seroconversión de Chagas, así como muestras de suero positivas y negativas provenientes de zonas endémicas de la enfermedad y muestras positivas de otras enfermedades que podían interferir con la prueba. Las pruebas Bioelisa CHAGAS, Chagatest ELISA recombinante v. 4.0, Chagatest HAI y SD BIOLINE CHAGAS Ab Rapid, se emplearon como referencia.

**Resultados.:**

Los porcentajes de sensibilidad y especificidad clínica fueron de 97,73 % (IC_95%_ 96,23-99,24) y 99,33 % (IC_95%_ 98,88-99,78), respectivamente. Se obtuvo un 98,96 % de eficacia y una buena precisión.

**Conclusiones.:**

Los resultados demuestran que la nueva fase sólida del UMELISA CHAGAS® puede utilizarse para el inmunodiagnóstico, la certificación de sangre y la vigilancia epidemiológica en países endémicos y no endémicos con población de alto riesgo.

## Abstract (en)

**Introduction:**

Most people with Chagas disease develop specific antibodies against *Trypanosoma cruzi.* In early infection, IgM antibodies against *T. cruzi* are produced and later replaced for IgG antibodies during the course of the disease. The first symptoms of the infection may be very mild and atypical, which is why the disease is often not detected in the acute phase.

**Objectives:**

To evaluate the clinical and analytical sensitivity, and specificity, accuracy, and efficacy of UMELISA CHAGAS™ with the addition of new synthetic peptides in the solid phase representative of the shed acute phase antigen protein (SAPA) and the trypomastigote surface antigen (TSA).

**Materials and methods:**

We evaluated a mixed anti-I *cruzi* titer performance panel and a Chagas seroconversion one, as well as positive and negative serum samples from endemic areas of the disease and positive samples for other diseases that may interfere with the assay. The Bioelisa CHAGAS assay, Chaga test recombinant ELISA v.4.0, Chagatest HAI, and SD BIOLINE CHAGAS Ab Rapid were used as reference tests.

**Results:**

The sensitivity of the assay was 97.73% (95% CI: 96,23-99,24) and the clinical specificity, 99.33% (95% CI: 98,88-99,78) while the efficacy and the accuracy were 98.96%.

**Conclusions:**

Our results show that the new solid phase of UMELISA CHAGAS^®^ can be used for immunodiagnostic, blood certification, and epidemiological surveillance in endemic and non-endemic countries with high-risk populations.

La gran mayoría de las personas afectadas por la enfermedad de Chagas desarrollan anticuerpos específicos contra *Trypanosoma cruzi.* En la etapa inicial de la infección se producen anticuerpos IgM, que son reemplazados por anticuerpos IgG a medida que progresa la enfermedad ^1^.

Las manifestaciones clínicas de la enfermedad en su etapa inicial pueden ser muy leves y atípicas, y producir solamente malestar general, motivo por el cual con frecuencia la enfermedad no se detecta en su fase aguda.

La proteína SAPA *(Shed Acute Phase Antigen)* se expresa principalmente en la forma infectiva de *T. cruzi,* lo que produce una temprana y fuerte reacción de la inmunoglobulina G después de la infección, la cual disminuye con el avance de la enfermedad ^2^. Los anticuerpos contra la SAPA están presentes fundamentalmente en el suero de pacientes en la fase aguda y en aquellos que adquieren la enfermedad de forma congénita ^3^^-^^5^. Además, la actividad trans-sialidasa de la molécula implicada en la invasión del parásito no ha sido detectada en *T. rangeli, Leishmania* spp. ni *Plasmodium* spp., parásitos que comparten la distribución geográfica de *T. cruzi*^6^^,^^7^. Por estas razones, es importante el uso de este antígeno en la fase sólida del UMELISA CHAGAS^®^, ya que permitiría la detección de anticuerpos en la infección temprana y evitaría reacciones inespecíficas con otros parásitos.

El antígeno TSA *(Trypomastigote Surface Antigen)* es uno de los blancos de los linfocitos T citotóxicos CD8+ en la infección humana y se caracteriza por tener en su secuencia hasta cuatro copias de un motivo de aminoácidos muy conservado en neuraminidasas bacterianas ^8^^,^^9^. En diversos estudios se ha reportado el uso de la proteína TSA para el diagnóstico de la enfermedad de Chagas, con buenos resultados en términos de sensibilidad y especificidad.

El UMELISA CHAGAS^®^ es una prueba inmunoenzimática indirecta en la cual la fase sólida está constituida por placas de tiras recubiertas con tres péptidos sintéticos monoméricos, el P-17, el P-18 y el C-12, correspondientes a regiones inmunodominantes de la membrana del parásito. Con la incorporación de los monómeros SAPA y TSA, así como la sustitución de los monómeros P-17 y C-12 por el quimérico Q-5 formado por estos, se pretende mejorar de forma cuantitativa los indicadores de sensibilidad, especificidad, precisión y eficacia que determinan el buen desempeño o funcionamiento de la prueba con una mayor representación de epítopos inmunodominantes de *T. cruzi.*

El propósito del estudio fue evaluar el UMELISA CHAGAS^®^ con la incorporación de nuevos péptidos monoméricos y quiméricos representativos de diferentes regiones de *T. cruzi.*

## Materiales y métodos

### Obtención de los péptidos sintéticos

Todos los péptidos se obtuvieron mediante la síntesis química en fase sólida descrita por Merrifield en 1963, siguiendo la estrategia tercbutiloxicarbonilo (BocJ en bolsas de polipropileno ^10^^-^^12^. Se sintetizaron tres péptidos: el péptido quimérico Q5, formado por repeticiones de los péptidos monoméricos P-17 y C-12 representativos de epítopos inmunodominantes de la membrana de *T. cruzi,* que son los utilizados en el UMELISA CHAGAS^®^ actual; el monómero SA-15, correspondiente a la región más antigénica de la proteína SAPA del parásito, y el péptido monomérico TSA-E3, que codifica para una parte del antígeno de superficie del tripomastigote.

### Muestras utilizadas en la evaluación

Se emplearon las siguientes: muestras de suero presuntamente negativas procedentes de donantes de sangre cubanos sanos del Banco Provincial de Sangre de La Habana (n=896); muestras de suero positivas (n=441) confirmadas por inmunofluorescencia indirecta (IFI) provenientes de diferentes países endémicos de la enfermedad de Chagas; muestras de suero negativas para la enfermedad de Chagas provenientes de zonas endémicas (n=591) evaluadas por IFI, y muestras de suero negativas para la enfermedad de Chagas y positivas para otras enfermedades que pudieran interferir en la prueba. Todas las muestras se evaluaron con las pruebas diagnósticas SUMA^®^ (UMELISA HCV, UMELOSA HCV UMELISA HBsAg PLUS, HBsAg confirmatory test, UMELISA HCV UMELISA Dengue IgM PLUS) según correspondiera.

Asimismo, se emplearon muestras de pacientes con hepatitis C (n=8), de individuos vacunados contra la hepatitis B (n=16), de pacientes con dengue (IgM) (n=12), de pacientes con hepatitis B (n=16), de suero positivas para el HTLV (n=16), y de pacientes en hemodiálisis atendidos en el Hospital Miguel Enríquez de La Habana (n=18).

Se utilizaron dos paneles comerciales: el de desempeño de título mixto anti*-Trypanosoma cruzi* (Chagas) PMT 204 (n=21) (Laboratorios Lincon, S.A., México), y el de serconversión de Chagas (I *cruzi)* AccuVert™ (0615-0038) (n=10) (Laboratorios Lincon, S.A., México).

### Características evaluadas de la prueba

Se evaluaron la sensibilidad y la especificidad clínica y analítica; se hizo la prueba de concordancia utilizando el índice kappa (K), así como un estudio de precisión mediante la determinación de la reproducibilidad y la eficacia de la prueba. Para el cálculo de estos indicadores, se elaboró una tabla de contingencia.

*Especificidad y sensibilidad clínicas.* La especificidad clínica se expresó como el porcentaje de negatividad en muestras en las que el analito de interés estuviera ausente y se calculó con los valores verdaderos negativos divididos por la suma de los verdaderos negativos más los falsos positivos ^13^. La sensibilidad clínica se expresó como el porcentaje de positividad en muestras en las que el analito de interés estuviera presente y se calculó como los valores verdaderos positivos divididos por la suma de los verdaderos positivos y los falsos negativos ^13^.

*Especificidad analítica.* El grado para distinguir entre el analito de interés y otros componentes en la muestra ^13^ se calculó evaluando muestras positivas para otras enfermedades que pudieran interferir en la prueba.

### Estudios de concordancia

La concordancia entre los diferentes métodos de referencia y el UMELISA CHAGAS^®^ frente a un mismo panel de muestras, se determinó mediante el índice de concordancia kappa (K), cuyo valor mayor de 0,6 denota una elevada concordancia ^13^ (cuadro 1).

**Cuadro 1:** Clasificaciones de concordancia según el índice kappa

Concordancia	Kappa
Deficiente	<0,20
Regular	0,21-0,40
Moderada	0,41-0,60
Buena	0,61-0,80
Muy buena	0,81-1,00

### Eficacia del ensayo

La capacidad de la prueba para detectar correctamente todos los positivos y negativos, se calculó como la suma de los valores verdaderos positivos más los verdaderos negativos divididos por la suma de los verdaderos positivos y los falsos positivos más los falsos negativos y los verdaderos negativos ^13^.

### Precisión

Esta se calculó en términos de reproducibilidad al determinar el grado de concordancia entre los resultados de una serie de réplicas (aproximadamente 20) de una misma muestra en diferentes días. Se analizaron dos muestras positivas en el estudio con grados de positividad alto (PA) y bajo (PB), una muestra falsa positiva (FN) y una negativa (N).

### Procedimiento

En la evaluación se utilizó una prueba UMELISA indirecta con reactivos certificados del UMELISA CHAGAS^®^ y el equipamiento de la tecnología SUMA^® (^^14^^-^^16^. Como referencias se emplearon las pruebas comerciales Bioelisa Chagas^®^ (BIOKIT, España) ^17^, Chagatest ELISA recombinante^®^, v. 4.0 (Wiener Lab, Argentina), Chagatest HAI^®^ (Wiener Lab, Argentina) ^18^ y SD BIOLINE CHAGAS Ab Rapid^®^ (Standard Diagnostics Inc., Korea) ^19^. Las muestras se consideraron positivas cuando resultaron reactivas en alguna de las pruebas de referencia.

### Análisis e interpretación de los resultados

En el análisis e interpretación de los resultados con un nivel de confianza de 95%, se utilizó el sistema Stripe Reader Software™, versión 9.0, para la automatización del trabajo con los lectores de tiras y estuches UMELISA de la tecnología SUMA^®^ (Cuba) ^14^^-^^16^^)^ y el programa para el análisis epidemiológico de datos tabulados Epidat, versión 3.1 (España). Las muestras se clasificaron en verdaderos positivos y falsos negativos tomando como referencia el resultado de las pruebas comerciales.

### Consideraciones éticas

El estudio cumplió con los principios establecidos en la Declaración de Helsinki "Recomendaciones que guían a los médicos en la investigación biomédica con seres humanos" adoptada por la XVIII Asamblea Médica Mundial (Helsinki, Finlandia, junio de 1964) y sus sucesivas enmiendas, y fue aprobado por el comité de ética de la institución en la que se llevó a cabo.

## Resultados

### Sensibilidad y especificidad

Se evaluaron 1.928 muestras de suero, de las cuales 10 resultaron falsas positivas y 10 falsas negativas con respecto a la prueba de referencia Chagatest HAI^®^, para 97,73 % (IC_95%_ 96,23-99,24) y 99,33 % (IC_95%_ 98,8899,78) de sensibilidad y especificidad clínicas, respectivamente. La especificidad analítica fue de 100 %.

### Eficacia de la prueba

La proporción de resultados válidos de la prueba fue de 98,96 %.

### Estudio de concordancia

La prueba de concordancia con el índice kappa (K) resultó ser de 0,97 (IC_95_% 95-98), es decir, una muy buena concordancia (cuadro 2).

**Cuadro 2:** Tabla de contingencia para determinar la sensibilidad y especificidad de la prueba

		Referencias	Total
		Positivos Negativos	
Prueba	Positivos	431 10	441
	Negativos	10 1.477	1.487
	Total	441 1.487	1.928

Sensibilidad: 97,73 % (IC_95%_ 96,23-99,24)

Especificidad: 99,33 % (IC_95%_ 98,88-99,78)

### Estudio de precisión

Los porcentajes de reproducibilidad fueron del 100 % en todos los casos, lo que indica la buena precisión de la prueba.

## Discusión

El desarrollo y la producción de antígenos naturales, recombinantes y sintéticos para el inmunodiagnóstico de la enfermedad de Chagas, constituyen el objetivo fundamental de varios trabajos de investigación. Sin embargo, en la actualidad los péptidos sintéticos se utilizan ampliamente por las ventajas que significan en las pruebas diagnósticas en que se utilizan ^12^.

El uso de péptidos sintéticos como antígenos en el inmunodiagnóstico, los cuales se diseñan para obtener una prueba que garantice resultados con gran sensibilidad y especificidad, constituye una solución al problema del diagnóstico serológico de la enfermedad de Chagas ^12^^,^^20^. Entre los péptidos sintéticos, los quiméricos permiten "simular" estructuras de la proteína nativa en algunas ocasiones. Por ello, en el presente estudio se sintetizaron péptidos quiméricos con estas características no descritos por otros investigadores, con lo cual nuestro grupo de trabajo obtuvo el certificado de autor de invención ^21^.

En la selección de las secuencias aminoacídicas para el diseño del péptido quimérico, se tuvieron en cuenta los resultados de los estudios de los péptidos monoméricos descritos en la literatura y los obtenidos por nosotros en el laboratorio ^22^.

El antígeno SAPA se ha propuesto para el diagnóstico temprano de la enfermedad de Chagas debido a que los anticuerpos contra este antígeno están presentes fundamentalmente en el suero de pacientes en la fase aguda y en aquellos que adquirieron la enfermedad de forma congénita ^3^^-^^5^.

En varios estudios se ha reportado el uso de la proteína TSA para el diagnóstico de la enfermedad de Chagas, con buenos resultados en términos de sensibilidad y especificidad. La TSA es uno de los blancos de los linfocitos T citotóxicos CD8+ en la infección humana y se caracteriza por tener en su secuencia hasta cuatro copias de un motivo de aminoácidos muy conservado en neuraminidasas bacterianas ^8^^,^^9^.

### Sensibilidad y especificidad clínicas

El porcentaje de sensibilidad (97,73 %) del UMELISA CHAGAS^®^ con la incorporación de estos antígenos en la fase sólida, fue similar al reportado por investigadores colombianos con los estuches comerciales Bioelisa CHAGAS^®^, Chagatest ELISA recombinante^®^, versión 3.0, Chagatest HAI^®^ y SD BIOLINE CHAGAS Ab Rapid^®^ en su estudio de comparación de la capacidad diagnóstica de siete métodos de determinación de la infección por *T. cruzi* en pacientes con enfermedad de Chagas crónica, en el cual obtuvieron valores de sensibilidad entre 95 y 98 % ^23^. En evaluaciones del kit ELISA Chagas IICS^®^, versión 1 ^24^^,^^25^, se reportaron resultados similares al obtenido por nuestro grupo de trabajo con la nueva modificación de la fase sólida.

Los resultados discordantes del presente estudio pudieran deberse a dos posibles causas: los distintos tipos de antígenos y principios utilizados en las pruebas de referencia ^26^ y la composición del conjugado que contiene anti-IgM además de anti-IgG humana, que puede generar falsos positivos por su baja especificidad ^27^.

Uno de los usos más importantes de las pruebas diagnósticas serológicas es en las zonas endémicas, donde estas deben discriminar a los individuos infectados de aquellos que, sin estarlo, pueden tener otras condiciones que generen falsos positivos. Para determinar la especificidad clínica, se evaluaron muestras negativas provenientes de regiones endémicas de la enfermedad y muestras de donantes cubanos de sangre supuestamente sanos, dado que la enfermedad no existe hoy en Cuba. La especificidad del UMELISA CHAGAS^®^ fue de 99,33 % (IC_95%_ 98,88-99,78) y en otras pruebas se han reportado resultados similares ^24^^-^^27^.

La especificidad analítica, parámetro que depende del principio de la prueba y del material que se investiga, resultó ser del 100 %, lo que indica que la incorporación de los nuevos péptidos sintéticos en el UMELISA CHAGAS^®^ permite detectar correctamente el analito de interés sin interferencia de ningún otro compuesto semejante.

El estudio de concordancia utilizando el índice kappa presenta diversas ventajas, destacándose su simpleza logística, la sencillez del análisis estadístico y una amplia aplicabilidad en la evaluación de métodos diferentes ^28^. En la práctica, cualquier valor de kappa mayor de 0,6 denota una buena concordancia. Un problema del uso de este índice es que sus valores dependen de la prevalencia de las muestras de cada categoría, lo que posibilita la comparación entre los diferentes índices utilizados en los estudios ^13^. En este caso, se obtuvo un valor de kappa de 0,97, lo que refleja muy buena concordancia entre el UMELISA CHAGAS^®^ con los nuevos péptidos sintéticos incorporados y las diferentes pruebas de referencia empleadas. En estudios sobre el desempeño del kit ELISA Chagas IICS^®^, versión 1, también se obtuvo una muy buena concordancia ^24^.

La eficacia o probabilidad de que un individuo sea clasificado correctamente por la prueba alcanzará su valor óptimo con aquella técnica que no arroje falsos resultados positivos y negativos ^13^. En el presente estudio, el UMELISA CHAGAS^®^ con la nueva modificación en su fase sólida obtuvo una eficacia de 98,96 %, con 10 muestras falsas positivas y 10 muestras falsas negativas. Las posibles explicaciones para los resultados falsamente negativos y positivos incluyen la presencia de diferentes antígenos (lisado natural, proteínas recombinantes y péptidos sintéticos) en las fases sólidas y la diversidad de metodologías empleadas en las pruebas de referencia ^23^.

Las principales limitaciones del estudio fueron la dificultad para adquirir muestras caracterizadas en las tres etapas de la enfermedad y las requeridas para determinar la reactividad cruzada con parásitos en estrecha relación filogenética con *T. cruzi,* así como las pruebas de referencia para una segunda evaluación de las muestras discordantes.

Los resultados de la evaluación del UMELISA CHAGAS^®^ con la incorporación de nuevos péptidos monoméricos y quiméricos representativos de diferentes regiones del parásito, demostraron su adecuado desempeño en la detección de anticuerpos contra *T. cruzi* en países endémicos y no endémicos para la enfermedad de Chagas. Estos resultados permiten recomendar su uso para el inmunodiagnóstico, la certiicación de sangre y la vigilancia epidemiológica en países endémicos y no endémicos con población de alto riesgo, así como para incorporar péptidos representativos de otras regiones inmunodominantes del parásito.
